# Immunomodulatory Role of Interferons in Viral and Bacterial Infections

**DOI:** 10.3390/ijms241210115

**Published:** 2023-06-14

**Authors:** Paulina Mertowska, Konrad Smolak, Sebastian Mertowski, Ewelina Grywalska

**Affiliations:** Department of Experimental Immunology, Medical University of Lublin, 20-093 Lublin, Poland; paulinamertowska@umlub.pl (P.M.); smolakkonrad93@gmail.com (K.S.); ewelina.grywalska@umlub.pl (E.G.)

**Keywords:** IFN-α, IFN-β, IFN-γ, immune system, bacterial infections, hepatitis C virus, Epstein–Barr virus, SARS-CoV coronavirus, herpes simplex virus

## Abstract

Interferons are a group of immunomodulatory substances produced by the human immune system in response to the presence of pathogens, especially during viral and bacterial infections. Their remarkably diverse mechanisms of action help the immune system fight infections by activating hundreds of genes involved in signal transduction pathways. In this review, we focus on discussing the interplay between the IFN system and seven medically important and challenging viruses (herpes simplex virus (HSV), influenza, hepatitis C virus (HCV), lymphocytic choriomeningitis virus (LCMV), human immunodeficiency virus (HIV), Epstein–Barr virus (EBV), and SARS-CoV coronavirus) to highlight the diversity of viral strategies. In addition, the available data also suggest that IFNs play an important role in the course of bacterial infections. Research is currently underway to identify and elucidate the exact role of specific genes and effector pathways in generating the antimicrobial response mediated by IFNs. Despite the numerous studies on the role of interferons in antimicrobial responses, many interdisciplinary studies are still needed to understand and optimize their use in personalized therapeutics.

## 1. Introduction

Interferons have been the subject of extensive research for several decades. In 1957, two scientists, Alick Isaacs and Jean Lindenmann, discovered interferons by observing a phenomenon called “interference” in viral replication. They found that virus-infected cells contain a soluble agent that can prevent the growth of other viruses, which was later identified as interferon. In the 1960s and 1970s, researchers discovered that interferons are proteins that have antiviral activity and can protect neighboring cells from viral replication. In the 1980s, researchers learned more about the structure and function of interferons thanks to protein isolation and purification methods. The US Food and Drug Administration (FDA) approved the first interferon-based therapy in the 1980s to treat hairy cell leukemia [1,2,3,4,5].

Further research in the 1990s and 2000s using molecular and genetic research led to a better understanding of interferons’ ability to regulate immune responses beyond antiviral activity. Interferons have been used to treat a variety of diseases, including chronic hepatitis B and C, certain cancers, and autoimmune disorders. Developments in recent years and the SARS-CoV-2 pandemic have contributed to the optimization of treatment protocols and deepening knowledge about the role of interferons in various diseases and immune responses [1,2,3,4,5].

These proteins activate the immune system’s defense mechanisms against various pathogens and are considered an essential component of the innate immune response [6,7]. They can activate immune system cells such as natural killer cells and macrophages, while also enhancing the speed of infection recognition by regulating antigen presentation to T lymphocytes. The human body can produce three different types of interferons, categorized based on the type of receptor they communicate with. Type I and III IFNs are induced in virtually all types of immune cells after recognizing viral components, while Type II IFN is restricted to T lymphocytes and NK cells and is induced by cytokines such as IL-12 [8,9,10,11,12,13,14,15]. Interferons not only strengthen healthy cells’ resistance to viral infections but also impair protein synthesis, effectively inhibiting virus multiplication. They also regulate and activate the immune response to bacterial infections [16,17,18]. This review presents an in-depth analysis of the characteristics and roles of the three types of IFN in viral and bacterial infections, including their mechanisms of action, molecular characteristics and signal transduction pathways.

## 2. Results and Discussion

### 2.1. Classification, Characteristics, and Mechanisms of Action of Interferons

Interferons, apart from interleukins and chemokines, belong to the family of cytokines, i.e., molecules regulating processes such as proliferation, differentiation, and cell motility. They are also mediators of the body’s defense reactions and participate in the regulation of hematopoiesis, which is why they are often referred to as hormones of the immune system with multifaceted antiviral and cell-modulating properties [19]. The research shows that interferons are mainly responsible for the activation of the immune system response, i.e., they have an immunomodulatory function, activity against a wide range of viruses, and regulation of cell growth and differentiation [20,21,22]. Currently there is a division of IFNs into three types based on affinity to a specific type of receptor, homology of amino acid sequences, and their functional properties.

#### 2.1.1. Type I IFNs

Currently, 21 types of IFN are known, of which 13 subtypes belong to type I IFN. This was confirmed by the team of Wu et al. [23] who showed that there are 13 subtypes of human IFN-α, where IFN-α1 and IFN-α13 share the same amino acid sequences (Table 1). They include IFN-α (alpha), IFN-β (beta), IFN-ɛ (epsilon), IFN-κ (kappa), IFN-ω (omega), IFN-ν (nu), IFN-τ (tau), subtypes IFN-δ (delta), IFN-ζ (zeta) and IFN-χ (chi). These were originally shown to be powerful antiviral immunoregulators, but recent research shows that they also play an important role in bacterial infections [12,24,25].

All types of type I IFN are similar in structure and functioning and they are distinguished from other interferons mainly by the type of stimulated receptor and unique physicochemical properties: the ability to maintain structural stability at 65 °C and pH 2 [26]. In terms of genetic organization, the genes responsible for encoding type I IFNs are located on chromosome 9, region 2, band 1 (9p21). In the human genome, type I IFNs are encoded by a 350 kb multi-gene family: thirteen non-allelic IFN-α genes, at least five pseudogenes, and single genes for interferon β, ω, κ, and ε [19,27]. To date, IFN-α, which is encoded by several genes, and IFN-β have been the best characterized and most extensively described [28]. IFN-ω and IFN-τ are also known, but they remain poorly characterized today due to limited tissue expression, overlapping functions with IFN-α and IFN-β, and species differences [18]. Type I interferon receptors are mainly expressed by T lymphocytes, macrophages, and monocytes [12] where they are induced in infected cells to produce an antiviral state in uninfected cells [18]. A characterization of type I IFN performed by our team showed that most IFN-α contains 52.79-61.78% of hydrophilic amino acids, while IFN-β 60.43%, IFN-ω 56.41%, IFN-ε 57, 69% and IFN-κ 60.39% (Table 1).

**Table 1:** Molecular characteristics of three types of interferons.

Types of IFNs	Subtypes	Gens	Protein Length (AA)	Molecular Mass (kDa)	Protein ID	PI	Amino Acid Composition		Secondary Structure		Reference
							% Hydrophilic	% Hydrophobic	α-Helix	β-Strand	
**Type I**	IFN-α1/13	*IFNA1; IFNA13*	189	21,725	P01562	5.17	57.14%	42.86%	6	0	[29]
	IFN-α2	*IFNA2*	188	21,578	P01563	6.05	57.14%	42.86%	2	0	[30]
	IFN-α4	*IFNA4*	189	21,808	P05014	5.54	58.20%	41.80%	5	0	[31]
	IFN-α5	*IFNA5*	189	21,942	P01569	5.48	57.14%	42.86%	5	0	[32]
	IFN-α6	*ITGA6*	1130	126,606	P23229	5.94	60.88%	39.12%	5	61	[33]
	IFN-α7	*CHRNA7*	502	56,449	P36544	5.73	52.79%	47.21%	12	16	[34]
	IFN-α8	*IFNA8*	189	21,989	P32881	5.17	57.14%	42.86%	5	0	[35]
	IFN-α10	*IFNA10*	189	21,835	P01566	5.75	57.67%	42.33%	5	0	[36]
	IFN-α14	*IFNA14*	189	22,063	P01570	6.49	56.61%	43.39%	5	0	[37]
	IFN-α16	*IFI16*	785	88,256	Q16666	8.42	61.78%	38.22%	15	25	[38]
	IFN-α17	*IFNA17*	189	21,728	P01571	5.50	57.67%	42.33%	5	0	[39]
	IFN-α21	*IFNA21*	189	21,741	P01568	6.07	57.14%	42.86%	5	0	[40]
	IFN-β	*IFNB1*	187	22,294	P01574	8.06	60.43%	39.57%	8	0	[41]
	IFN-ω	*IFNW1*	195	22,319	P05000	8.37	56.41%	43.59%	5	0	[42]
	IFN-ɛ	*IFNE*	208	24,414	Q86WN2	7.91	57.69%	42.31%	6	0	[43]
	IFN-κ	*IFNK*	207	25,218	Q9P0W0	7.66	60.39%	39.61%	7	0	[44]
**Type II**	IFN-γ	*IFNG*	166	19,348	P01579	8.74	63.85%	36.15%	7	0	[45,46]
**Type III**	IFNλ1/IL-29	*IFNL1*	200	21,898	Q8IU54	8.09	52.50%	47.50%	5	0	[47]
	IFNλ2/IL-28A	*IFNL2*	200	22,288	Q8IZJ0	7.13	52.00%	48.00%	6	0	[48]
	IFNλ3/IL-28B	*IFNL3*	196	21,706	Q8IZI9	7.52	52.04%	47.96%	6	0	[49]
	IFN-λ4	*IFNL4*	179	19,675	K9M1U5	11.17	50.84%	49.16%	4	0	[50,51]

This group is also characterized by a high diversity of amino acid sequences, ranging from 5.36% to 95.77% similarity (Supplementary Materials Table S2). In addition, analysis of amino acid sequences showed that three subtypes of type I IFNs are characterized by the presence of several isoforms resulting from alternative splicing. These include IFN-α6 in eight additional isoforms; IFN-α7 present in three additional isoforms and IFN-α16 present in four additional isoforms (Table 2).

#### 2.1.2. Type II IFN

The role of IFN type II (IFN-γ) as a key antiviral cytokine was originally discovered in a study of cell resistance to viral infections [52]. IFN-γ plays an important role in the innate and adaptive immune responses against bacteria, cancer, viruses, and pathological inflammatory processes [53]. IFN-γ is produced by various cells of the immune system, including natural killer cells, CD4+ helper T cells, and CD8+ cytotoxic T cells [12,54,55]. IFN-γ is encoded by the IFNG gene, located on chromosome 12 (12q15) [56]. Amino acid sequence analysis showed it consists of 63.85% hydrophilic amino acids (Table 1). IFN-γ is the only type II interferon and is serologically distinct from type I interferons; it is unstable in acid, while type I variants are stable in acid. In addition, analysis of the amino acid similarity of IFN-γ with other types of IFN showed it had relatively low similarity, ranging from 8.79% to 21.28% (Supplementary Materials Table S2). IFN-γ is both an important autocrine signal for APCs in the early innate immune response and an important paracrine signal in the adaptive immune response. IFN-γ expression is induced by the cytokines IL-12, IL-15, IL-18, and type I IFN [57]. In addition, IFN-γ induces the synthesis of other cytokines including interleukin 2, interleukin,6 and tumor necrosis factor TNF-α. Another function of IFN-γ is to stimulate the immune response by activating B and T lymphocytes. The antiviral activity of IFN-γ consists of blocking the synthesis of viral proteins and inhibiting the multiplication of virions. In addition, interferon-gamma enhances the expression of MHC class I and II molecules. This cytokine also has an antiproliferative effect, so it can limit the proliferation of cancer cells. The pro-inflammatory activity of gamma interferon consists of inducing macrophages and endothelial cells [58,59].

#### 2.1.3. Type III IFN

Type III IFNs (IFN-λ) are the least characterized family of interferons involved in the innate immune response. They are structurally related to type I IFN and to the IL-10 family of interleukins [60]. They collectively induce an antiviral state in cells by inducing the expression of INF-stimulated genes (ISGs). Type III IFNs play an important role in the antiviral defense of the mucosal epithelium, which makes type III IFNs the primary factor in the first line of defense against viral infection in places of external contact with the pathogen [61]. According to the literature, the IFN type III group currently includes four subtypes: IFN-λ1 (IL-29), IFN-λ2 (IL-28A), IFN-λ3 (IL-28B), and IFN-λ4. They differ in their biochemical properties, amino acid composition (Table 1) and the degree of amino acid sequence identity with other types of IFN, with values ranging between 7.02% and 95.92% (Supplementary Materials Table S2). Detailed amino acid sequence analysis revealed that IFN-λ4 exists in four additional alternatively spliced isoforms (Table 2).

In type III IFNs, the production and expression of IFN-λ4 are controlled by the double nucleotide polymorphism IFNL4 rs368234815 (ΔG/TT), and the IFN-λ4 protein is produced by individuals carrying a functional genetic variant of the IFNL4-ΔG allele [62].

### 2.2. The Role of the Signal Transduction Mechanism in the Activity of Interferons

In the human body, there are several mechanisms involved in the innate immune response that protects against pathogens, particularly viruses. One such mechanism is the pattern recognition receptors (PRRs), which detect specific molecular patterns associated with pathogens. These PRRs play a crucial role in initiating the antiviral immune response.

To initiate the antiviral response, PRRs must first detect pathogen-associated molecular patterns (PAMPs) [6,63,64]. Toll-like receptors (TLRs), retinoic acid-induced gene-like receptors (RLRs), and Nod-like receptors (NLRs) are examples of PRRs. These receptors are located on the surface of cells and within cells, and when they detect PAMPs, they activate signaling pathways that result in the production and release of interferons. Researchers are paying particular attention to pattern recognition receptors (PRRs), which detect their presence and bind to pathogen-associated molecular patterns (PAMPs) and damage-associated molecular patterns (DAMPs) [6,63,64] The former group includes viral proteins, viral DNA, and viral RNA, while the latter group includes products resulting from virus-related cell damage and death [65]. Activation of PRR triggers the initiation of IFN production in the cell, which, upon binding to the corresponding receptor, initiates a signal transduction cascade resulting in ISG activation [66,67]. ISG activation induces an antiviral response in the cell. One of the best examples of PRR action is the signaling pathway mediated by Toll-like receptors 3 (TLR3), which, when activated by the presence of HSV, triggers the activation of a MyD88-independent signaling pathway (via the Toll/IL1 receptor domain containing the IFNβ inducing adapter (TRIF) and TRAF3), causing IRF3/7 to be translocated into the nucleus to stimulate type I IFN production. TLR3 activation also triggers the production of inflammatory cytokines and chemokines (via TRIF and TRAF6 activation of NF-κβ) [68].

Other studies have implicated RIG-I-like receptors in pathogenesis, which researchers believe are a different class of viral dsRNA sensors and include molecules such as MDA5 and RIG-I. Upon their activation, they bind to the mitochondrial antiviral signaling protein (MAVS), which activates the IRF3/7 and NF-κβ signaling pathways, resulting in increased production of type I and type III IFNs [69]. Another example of the use of the IRF3/7 signaling pathway for the production of type I IFN is the activity of cGAS, which is a DNA sensor that allows it to bind to viral dsDNA. According to researchers, such a combination induces the synthesis of 2’3’-cGAMP, which after binding to a protein associated with the endoplasmic reticulum, the stimulator of IFN genes (STING) signals through TANK 1 (TBK1) binding kinase, resulting in activation of IRF3/7 [70,71]. As a response to PRR activation, type I interferons, including IFN-alpha and IFN-beta, are produced and released by infected cells and bind to specific receptors on neighboring cells. This binding triggers a cascade of antiviral defense mechanisms that help prevent the spread of infection.

**Figure 1:**
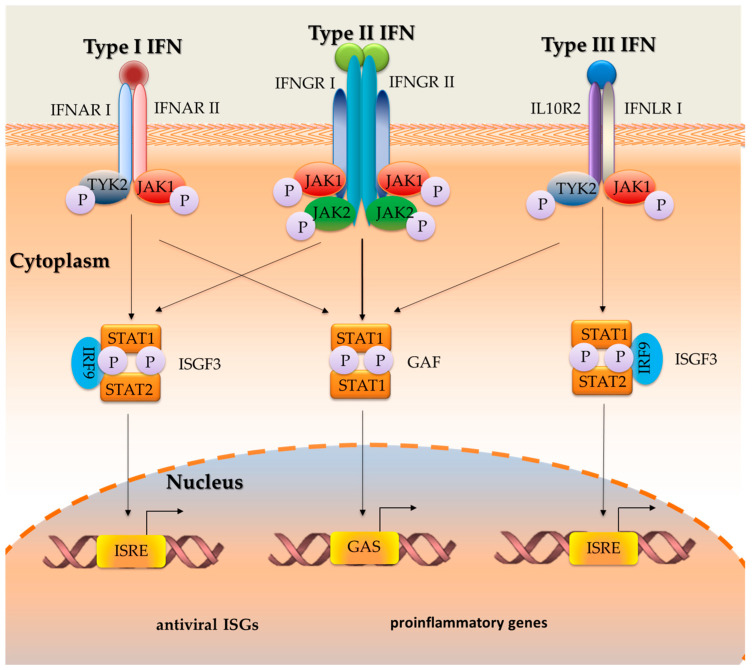
Mechanisms of signaling in the cell by different types of interferons (based on [6,77]). IFN—interferon; IFNAR—IFNA receptor; IFNAR1—IFNα receptor 1; IFNAR2—IFNα receptor 2; IRF—regulatory factor; ISG—IFN stimulated gene; JAK-STAT—Janus kinase/signal transducer and activator of transcription; STAT1—signal transducer and activator of transcription 1; STAT2—signal transducer and activator of transcription 2; TYK2—tyrosine kinase 2; GAF—IFNγ activation factor; ISGF3—IFN-stimulated gene factor 3; IRF9—IFN-regulatory factor 9; ISRE—IFN-stimulated response elements; IFNGR—IFNγ receptor; IFNGR1—IFNγ receptor 1; IFNGR2—IFNγ receptor 2; IFNLR1—IFNλ receptor 1 (also known as IL-28Ra) and IL-10R2 (also known as IL-10Rβ); GAS—gamma-activated sequences; P—phosphorus.

Furthermore, data show that all types of IFN have a separate heterodimeric receptor on the surface of each cell. The structural similarity between type I IFN subtypes is limited, but all share the same heterodimeric IFNA receptor (IFNAR) consisting of the high-affinity receptor IFNAR2 and the low-affinity receptor IFNAR1 [51]. IFNs type II bind to the IFNGR receptor complex (composed of IFNGR1 and IFNGR2), and type III IFNs bind to the IFNLR receptor complex (composed of IFNLR1 and IL-10Rβ), which in turn binds to the Janus kinase receptors JAK1 and JAK2. Activation of these kinases then leads to STAT1 homodimerization, nuclear translocation, and binding to gamma-activated sequences on DNA [72]. Type I and type III IFNs bind to different heterodimeric receptors, activating similar signaling pathways and transcriptional responses (Figure 1) [73]. The binding of type I, II and type III IFNs to their respective receptor complexes results in cross-phosphorylation of JAK1-activated kinase and tyrosine kinase 2 (TYK2) via the cytoplasmic domains of the receptor subunits [54]. However, it has recently been shown that JAK2 can also be phosphorylated by type III IFN but not by IFN type I [74,75]. Following the cross-phosphorylation process, the antiviral signal is transduced by a transcription activator (STAT). This then triggers the phosphorylation of IFN regulatory factor 9 (IRF9) to form IFN 3 (ISGF3) complexes or the phosphorylation of IFN-γ activation factor (GAF), which consists of STAT1 and STAT2 homodimers. These complexes are then translocated to the nucleus where they bind response elements stimulated by IFN regulatory factor (ISRE) or gamma ray sequences (GAS) on gene promoters stimulated by transcription activation of IFN (ISG) and (GAF), causing a response to viral infection [72,76]. Individual binding to these promoter elements results in the transcription of hundreds of genes involved in the antiviral response, including ISG, IFN, IRF, and STAT, OAS (oligoadenylate synthases); GBP (guanylate binding proteins); NOS2 (nitric oxide synthase 2); IFITM (IFN-induced transmembrane proteins); and TRIM (triremembered motif proteins) [2].

Although type I and type III IFNs belong to the innate immune response and show clear similarities in signaling and biological functions, both types of IFNs show differences that affect their biological functions. Type I IFN plays a pathogenic role in autoimmune diseases and is widely expressed in virtually every cell type, whereas IFNLR1 expression is restricted, mainly to epithelial cells lining mucosal surfaces.

Furthermore, while all cells are capable of expressing type I IFN, particularly when infected with a virus, only certain subsets of cells, again particularly epithelial cells, are capable of expressing type III IFN. Therefore, type III IFN is considered to be of paramount importance as a first-line and local innate defense against viral pathogens. The conducted studies do not fully clarify how IFNLR1 expression is limited to cells of epithelial origin, but there are indications that cell type-dependent epigenetic changes of the IFNLR1 gene, including DNA methylation and histone modifications, may be involved [75]. Indeed, DNA methyltransferase and histone deacetylase (HDAC) inhibitors have been reported to cause IFNLR1 gene silencing, increasing receptor expression and restoring sensitivity to IFN-λ in previously unresponsive cells, thereby increasing protection against viral pathogens, including herpes simplex 1 (HSV1) [78]. It now appears that activation of more than one signaling pathway is required to produce different biological properties of IFNs, and the signaling cascade alone is not sufficient to generate any given biological endpoint [74,79].

According to data in the literature, it is estimated that the effects of interferons are mediated by the induction of approximately 2000 ISG gene products. However, according to researchers, most of these ISGs do not directly initiate immunity to viral infection but rather influence the induction of signaling molecules that are responsible for stimulating immune responses to pathogens and disseminating signals received from infection sites [80,81,82,83]. Scientific studies indicate that there are four main effector pathways of the IFN-mediated antiviral response: the Mx antiviral protein pathway, the 2’,5’-oligoadenylate synthetase targeting ribonuclease L pathway, the R protein kinase pathway, and the ISG15-like pathway [2] (Table 3).

**Figure 2:**
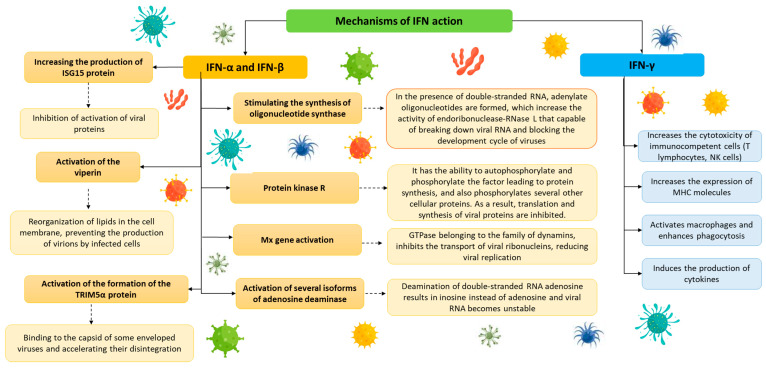
Scheme of the antiviral effect of interferons in a cell (based on [55,96,97]).

By influencing the activity of other cells, interferons contribute not only to the formation of antiviral factors but also to a specific type of antimicrobial readiness, involving many signaling pathways and activation of the body’s immune mechanisms. Activation of selected pathways causes modulation of various parts of the immune response, including non-specific mechanisms (activity of cytotoxic cells, production of complement proteins), the activity of B-line cells (responsible for the production of antibodies), and the expression of class I and II histocompatibility antigens. Interferons α and β are much more potent antivirals than IFN-γ; however, all types of interferons are necessary to activate the body’s full defenses against viruses (Figure 2).

Research is currently underway to identify and elucidate the precise role of individual genes and effector pathways in the generation of interferon-mediated antimicrobial responses. The effector pathways have been shown to block viral transcription, degrade viral RNA, inhibit translation, and modify protein function to control all steps of viral replication. Ongoing research continues to reveal additional activities of these effector proteins and unexpected antiviral response functions [2].

### 2.3. The Role of Interferons in the Course of Viral Infections

The effect of IFN on virus-infected cells and surrounding tissues results in increased expression and antiviral activity of IFN-stimulated genes in the host organism [98]. However, viruses have developed several anti-host mechanisms to promote efficient viral replication, thereby minimizing the antiviral power of IFN [99]. Such mechanisms include the activation of pathways that control IFN signaling, blocking the function of IFN-stimulated gene products, and interfering with various levels of intercommunication between IFN and other cellular pathways [27].

In this review, we will focus on discussing the interrelationships between the IFN system and seven medically important and challenging viruses (Lymphocytic Choriomeningitis Virus (LCMV), hepatitis C virus (HCV), Immunodeficiency Virus (HIV), Epstein–Barr virus (EBV), SARS-CoV coronavirus, influenza and herpes simplex virus (HSV)) to highlight the diversity of viral strategies. Understanding the complex cellular network of antiviral processes and virus–host interactions should help identify new targets for therapeutic intervention in viral infection [10].

#### 2.3.1. Herpes Simplex Virus (HSV)

The herpes simplex virus (HSV) is one of the most widespread viruses in the world population, and humans are its only natural reservoir [98,99]. Epidemiological data indicate that its dissemination in the environment is possible only through direct contact with infected secretions [100,101]. The literature indicates that there are two types of herpes virus (1 and 2), both of which belong to the *Herpetiviridae* family. In addition to HSV viruses, this family also includes cytomegalovirus (CMV), herpes zoster (VZV), mononucleosis (EBV), human herpesvirus type 6 (HHV-6), human herpesvirus type 7 (HHV-7) and simian herpesvirus type B (HBLV). A characteristic feature of these viruses is their ability to go into latency and undergo reactivation. In clinical terms, HSV infection can take many forms, ranging from asymptomatic infections, through mucocutaneous infections and keratitis, to the most severe, highly lethal inflammations of the central nervous system [102]. Clinical reports indicate that in immunocompetent individuals, reactivation usually occurs within the anatomical innervation and in the vicinity of one dorsal ganglion, and if the primary infection occurs with the first to fourth decades of life, reactivation can occur at any time of the patient’s life [100]. Scientists are still looking for answers regarding the functioning of the mechanisms responsible for the entry latency and reactivation of HSV viruses. One of the demonstrated mechanisms involved in these processes, according to the research, may be granzyme B secreted by CD8+ cells, which degrades the virus ICP-4 protein necessary for its reactivation, keeping it in latency [103,104,105]. Many studies emphasize the important role of the innate immune system in the pathogenesis of HSV infections.

The development of this topic in the context of the role of interferons in response to HSV infection is supported by research conducted by You’s team in 2020. They demonstrated that β-catenin is an important component of the cGAS-STING signaling pathway, and blocking the nuclear translocation of β-catenin resulted in a decrease in the production of type I IFN [106].

In addition, the US3 protein kinase antagonizes IFN production by targeting β-catenin. According to the researchers, these findings shed new light on the mechanisms by which HSV-1 evades host antiviral immunity and will contribute to a better understanding of the interaction between host and HSV-1 infection [106]. Genetic studies show that viral modulation of IFN secretion, in particular by PKR, is the main basis of neurovirulence and pathogenesis associated with HSV infection [107]. In the literature, it is stated that PRR is also classified as ISG, although according to the researchers, they are always present in cells at a basic level of expression, and their expression is increased in response to IFN. This enhances the ability of host cells to recognize HSV PAMPs and thereby enhance IFN responses and the antiviral status mediated by TLR2, TLR3, TLR4, TLR7, cGAS, IFI16, MDA5, RIGI-I and PKR [108,109].

Further investigation of the role of interferon in the pathogenesis of HSV infections will focus on the study of keratinocytes, which researchers believe are the main source of IFN-β, and plasmacytoid dendritic cells (pDCs) infiltrating the dermis as a source of both IFN-α and IFN-β [110,111,112]. According to the literature, pDCs infiltrate the dermis after infection and it is suggested that they interact with other cells of the immune system to control viral replication [113]. Research conducted by Li’s team in 2020 showed that IFN-κ in vitro can be secreted by human keratinocytes [114]. This finding is extremely significant because the researchers showed that blocking the expression of IFN-κ contributes to an increase in HSV-1 replication. This allows us to hypothesize that IFN-κ may play an important role in controlling HSV-1 in herpes lesions [115,116].

Further analysis of the role of interferons in the pathogenesis of HSV showed that type I IFN activates natural killer cells that produce IFN-γ, which plays an important protective role against both HSV-1 and HSV-2 infections [115]. In addition to DCs, CD4+ and CD8+ T cells also contribute to IFN-γ production and also infiltrate the dermis [114]. Research conducted by Kim’s team in 2015 explains that this may be due to the migration of HSV-1-infected cells from the epidermis to the dermis, where they undergo apoptosis and then are taken up by skin DCs, presenting the antigen to CD8+ T cells [116]. This is also confirmed by research by the Harpur team in 2019, which showed that skin DCs and conventional DCs can work together to produce a suitable CD4+ T cell response [117]. Research by Zhu et al. in 2007 and 2009 showed that a subset of HSV-2-specific CD8+ T cells persist in the body long after the virus has been cleared from the lesion. According to these researchers, these lymphocytes may be involved in immune surveillance to control herpes infection [118,119]. Studies have indicated that macrophages play a role in producing IFN-α and IFN-β during HSV infection. As the infection progresses, these cells also contribute to the increase in IFN-γ production by natural killer cells through the secretion of IL-12 [120]. The combination of IFN-γ and IFN-α/β secreted at the site of infection may act synergistically both in vitro and in vivo, which contributes to the reduction in HSV-1 replication [121,122].

Other studies conducted by Peng et al. indicate that the basic mechanisms of interaction between IFN-β1 and IFN-γ function as an independent mechanism to induce separate categories of genes or as a cooperative mechanism (due to the induction of a common set of genes). Researchers showed that genes that were involved in the co-induction of both IFN subtypes led to apoptosis, triggering an inflammatory response and RNA degradation, which resulted in the inhibition of HSV-1 gene expression and increased DNA replication [123].

#### 2.3.2. Influenza Virus

Influenza is a highly contagious disease, making it very important to isolate the sick person as soon as possible. Despite the use of one name, several types of influenza can be distinguished in clinical practice. Depending on the type of virus that causes the disease, influenza type A, B, C and D are mentioned. Depending on the type of virus, the course of influenza is slightly different, and the symptoms, contagiousness, risks, and complications of the disease may also be different. All influenza viruses belong to the family *Orthomyxoviridae* (ssRNA) [124,125]. According to the data presented by Thompson et al. [126], there are about five million clinical cases of influenza each year, and the highest percentage of deaths is reported in the age group above 65 years. The first line of defense and the way to limit the spread of the influenza virus in the human body are the mechanisms of the innate immune response, both physical barriers and the production of the cytokines IFN and ISG by cells [127,128].

Among the many ISGs activated in influenza infection, the most important are genes from the GTPase Mx family (contributing to the inhibition of nuclear import and replication of viral nucleocapsids) [127,128,129], viperin (which reduces the release of viral particles from infected cells) [130] and IFITM3 (a member of the IFITM family involved in interfering with the fusion between viral and endosomal membranes, thus limiting virus entry) [131,132]. As in the case of HSV, PRRs also play an important role here. There are two main types of PRR playing a role in influenza pathogenesis by inducing IFN [133]. The first is TLR7 expressed on pDC, which recognizes ssRNA molecules as well as incoming virions in endosomal compartments, resulting in the production of high levels of type I IFN [134,135]. Researchers showed that TLR7 is only essential in DC cells as its expression on other immune cell subsets is not required for IFN induction [136,137,138,139]. Therefore, the second important PRR is TLR3, which is responsible for dsRNA recognition due to influenza virus infection [140,141]. Although this receptor is also involved in increasing IFN production in response to other viral infections, in the case of influenza virus it acts by activating pro-inflammatory signaling pathways [140,141]. Moreover, research conducted by Kato et al. and Loo et al. showed that the induction of IFN in response to influenza virus infection is mainly mediated by RIG-I, although these researchers believe that MDA-5 may also be involved in this process as its deficiency in infected cells resulted in a slight decrease in IFN induction and ISG responses [142,143]. However, the influenza virus has developed multiple strategies to escape host immune surveillance to achieve successful replication without IFN induction [125]. One such mechanism is the avoidance of recognition by PRRs. This is possible due to the nuclear nature of replication and transcription of this virus (unlike other RNA viruses that cycle in the cytoplasm). This means that cRNAs and progeny vRNAs, which include RIG-I and MDA-5, are generated in a location inaccessible to PRRs [144]. Another possible mechanism is that influenza virus encodes antagonists of the IFN system [145]. The NS1 protein is the primary antagonist which targets various components of the IFN induction cascade [146,147]. It can reduce RLR activation by sequestering dsRNA, inhibiting RIG-I activation in TRIM25 [148,149,150], and by interfering with processing and nuclear export of cellular mRNAs [151,152].

Innate immune responses, including IFN, play a key role in determining the pathogenicity and outcome of influenza virus infection: effective activation of the IFN system early in the infection effectively restricts viral replication and eliminates the virus, while over-activation of innate immune responses increases host tissue damage [153].

#### 2.3.3. Hepatitis C Virus (HCV)

HCV (Hepatitis C Virus) is a virus that causes hepatitis C. Modern medicine also knows other viruses that cause hepatitis (e.g., HBV, HAV, HEV). A typical feature of infections caused by hepatotropic viruses is damage to the liver parenchyma; however, HCV infection, unlike other infections, in most cases proceeds without characteristic symptoms and may manifest itself after many years in the form of cirrhosis or hepatocellular carcinoma [154]. Epidemiological data estimate that over 58 million people worldwide are infected with HCV, and there are about 1.5 million new infections each year [155,156]. Seven HCV genotypes (1-7) have numerous subtypes that differ in nucleotide sequence [157,158]. The HCV virus can evade the body’s defense mechanisms, including both innate and acquired immune mechanisms, resulting in a wide spectrum of pathogenicity, including the development of hepatocellular carcinoma [159].

The current gold standard of treatment for hepatitis C is weekly administration of pegylated IFN-α along with daily oral ribavirin (RBV) for 24 to 48 weeks. However, as research shows, this therapy is only effective in HCV 2 or 3 patients, causing sustained virological response (SVR) to develop in approximately 80–90% of treated patients. When infected with HCV genotype 1 or 4, only about half of patients can achieve SVR [160]. Clinicians have observed that there is a biphasic decline in HCV RNA levels in HCV infections treated with IFN therapy. This is observed after 8–9 h, which reflects the pharmacokinetics/pharmacodynamics of IFN, as well as the characteristics of the life cycle of HCV [161,162]. In addition to the viral genotype, the researchers also suggested that other factors such as gender or ethnicity are involved in the response to IFN therapy, which significantly indicates the involvement of specific types of genetic factors crucial for viral clearance [163,164]. Genetic studies, including genome-wide association studies, have shown a relationship between the occurrence of single nucleotide polymorphisms (SNPs) in the vicinity of the IFNL3 gene encoding IFN-λ3 and HCV infection and response to therapy. Research to date has focused on four SNPs: rs12979860 and rs8099917 for IFN-λ3, and rs368234815 and rs117648444 for IFN-λ4. Most of the research concerns the first two polymorphisms. The research team of Ge et al. showed that in the case of rs12979860 SNP, the favorable allele (CC genotype) was overrepresented in the European population in the group of people responding positively to interferon treatment, while the unfavorable TT genotype was more common in the African American population [165]. In addition, the researchers showed that the IFN-λ3 rs12979860 genotype was a better predictor of treatment outcome than assumed ethnicity because African Americans with the CC genotype had significantly more SVR than Europeans with the TT genotype. This theory was also confirmed by two other studies for the Australian and Japanese populations [166,167]. 

However, despite many studies, the exact mechanisms underlying the role of IFN-λ polymorphisms in HCV clearance are not well understood and require further intensive research. As in the case of other HCV viruses, it has also developed defense mechanisms against the immune response of the host organism. HCV can inhibit IFN production by interfering with the JAK-STAT pathway as well as blocking ISG mRNA translation and inhibiting the function of individual ISGs [168,169,170]. The most important mechanism that weakens the immune response is the blockade of translation induced by HCV by PKR phosphorylation and dimerization [171,172]. Phosphorylated PKR phosphorylates the eukaryotic translation initiation factor 2α-GDP (eIF2α-GDP) and prevents it from acting as a substrate for the eIF2B enzyme, which converts eIF2α-GDP to eIF2α-GTP, a key factor in protein translation [173]. ISG production and a translational block mediated by PKR are the mechanisms by which HCV significantly attenuates the interferon response [174]. Studies by Frese et al. [175] showed that MxA inhibited HCV RNA replication and the antiviral activity of IFN-α [175,176]. Another team (Itsui et al., [177]) found that overexpression of some ISGs, including MxA, significantly suppressed the HCV replicon, demonstrating the antiviral activity of MxA against HCV. Therefore, research data indicate that exogenous MxA protein may be involved in enhancing IFN-α and IFN-β activity in chronically HCV-infected patients by inhibiting HCV replication in the JAK-STAT signaling pathway [176]. 

#### 2.3.4. Lymphocytic Choroid Meningitis Virus (LCMV)

Another virus discussed is the lymphocytic meningitis virus (LCMV), which is a member of the *Arenaviridae* family. This virus causes lymphocytic meningitis (LCM), an infectious disease transmitted by rodents [178]. According to epidemiological data, the main host of LCMV is the house mouse, of which about 5% in the United States are carriers. Moreover, research reports indicate that the degree of infection of mice with this virus is geographically dependent. In addition to mice, cases of LCMV infection from hamsters and rodents in homes or in pet stores are also increasingly common [179,180]. According to the CDC, the prevalence of LCMV antibodies in human populations ranges from 2% to 5% [178]. 

Two signaling pathways are involved in LCMV recognition: the first via the TLR and the second via the RIG-I (RLR). Activation of these two signaling pathways is dependent on their expression on immune cells, with TLRs found mainly on macrophages and DCs, while RLRs are widely expressed on most cell types [181]. In the case of LCMV, the virus enters the cell via the endosome, where it is detected by TLR7 and TLR8, which recognize ssRNAs, and membrane-associated TLR2 is also involved in the response to LCMV. In addition to the pro-inflammatory response, TLR2 has been shown to contribute to the production of IFN-I, with TLR2-deficient mice having significantly reduced serum IFN-I bioactivity after infection with LCMV-WE [182]. Researchers also indicate that TLRs expressed on the pDC endosome are also capable of signaling through an additional unique pathway allowing for the production of large amounts of type I IFN. This is possible due to signaling through MyD88 and the IRAK-1 and IKKα kinases which phosphorylate the constitutively expressed factor transcriptional IRF7 that stimulates the expression of type I IFN [183,184,185]. The RIG-I protein is responsible for detecting nucleic acids in the cytosol that are formed as part of the LCMV replication cycle. Upon binding of nucleic acids to RIG-I, ATPase/helicase activity is stimulated, which exposes the caspase recruitment domain (CARD) that in turn binds to the adapter protein, mitochondrial antiviral signaling protein (MAVS). As a result of these processes, a signaling complex containing TRAF3, TBK1, and IKKε is formed, which activates the IRF3 transcription factor responsible for inducing the expression of early types of IFN-I and IFN-β, as well as IFN-α4 [186,187,188]. Multiple studies in mice have shown the importance of type I IFN in controlling LCMV infection. Research data indicate that loss of IFN-I signaling by blocking or deleting the IFNAR receptor may result not only in chronic infection but also in increased susceptibility to slowly replicating strains of LCMV [185,189,190,191]. This virus will also develop specific mechanisms of resistance to host IFNs. One such example is the LCMV Arenavirus nucleoprotein (NP), which blocks the nuclear translocation and transcriptional activity of the regulatory factor IRF-3, resulting in a strong inhibition of type I IFN production. This allows LMCV proliferation to be controlled due to abnormalities in innate immune response pathways [188,189,190,191,192,193].

According to data published by Martinez-Sobrido L. et al., the persistence of LCMV does not prevent type I IFN signaling, but rather suppresses endogenous IFN-β production in response to infection. These data also show that NP is the only LCMV gene product responsible for IFN antagonist activity in LCMV-infected cells and indicate that LCMV-NP can inhibit IRF-3-dependent transcription [194]. Expanding our knowledge on the mechanisms underlying the anti-IFN activity of LCMV NP will contribute to a better understanding of the pathogenesis, and immunogenesis of arenavirus infection and would facilitate the production of recombinant LCMV virus.

#### 2.3.5. Human Immunodeficiency Virus (HIV)

The most effective treatment regimen for the human immunodeficiency virus (HIV) is highly active antiretroviral therapy (HAART) to reduce HIV replication to undetectable levels. IFNs are used as powerful antiviral drugs that can activate the antiviral state in the body during HIV infection. Nevertheless, there is controversy as to whether IFN is an effective treatment for HIV due to its positive effect on viral infection and negative effect on chronic infection. Therefore, the exact role of type I IFN remains unclear and debated [195,196].

Studies by Cheng et al. showed that combination antiretroviral therapies (cART) inhibited HIV replication but failed to completely stop the elevated expression of ISGs, suggesting a prolonged induction of IFN type I [197]. cART stops the virus from making copies of itself in the body, which may result in slowing down the development of Acquired Immune Deficiency Syndrome (AIDS) [198,199]. Other research studies by Zhen A. et al. have shown that elevated type I IFN signaling during chronic HIV infection is a major cause of immune activation, inflammation, and depletion of cytotoxic T (CD8+) lymphocytes. This study confirms that IFN during HIV infection drives sustained immune activation and virus spread [200]. Although treatment with type I IFN is usually necessary to eradicate HIV, several mechanisms have evolved to bypass or suppress the effects of IFN in many situations. The research presented by Gargana et al. in 2018 showed that the mechanisms driven by many accessory proteins that the HIV uses to inhibit the therapeutic and endogenous effects of IFN-α result in a decrease in control over HIV infection [201]. Researchers also found that the HIV protein Vif plays a key role in the process of enzymatic post-translational protein modification (ubiquitination), self-catalysis, and proteosomal degradation of STAT1, STAT3 and monocyte cell lines in the canonical JAK-STAT pathway. All of the above processes allow HIV to inhibit the antiviral effects of type I IFN by inhibiting Vif-mediated STAT1 and STAT3, which consequently reduces the induction of the ISG-15 protein by IFN-α. Multiple mechanisms, which may be cell-specific, may explain the therapeutic failure of type I IFN in the course of HIV infection [201,202,203]. In addition, HIV can block the induction of type II and III IFNs in human dendritic cells and macrophages as a result of inhibition of the phosphorylation process of TANK1 binding kinase (TBK1) [204]. The HIV proteins Vif and Vpr have been shown to bind to TBK1 kinase, leading to inhibition of TBK1 trans-autophosphorylation, subsequent IRF3 phosphorylation, and induction of type I and III IFNs [205,206,207]. In addition, the HIV envelope protein (Gp120) has also been shown to play an important role in the inhibition of IFN-α, which is released and produced during viral infection of pDC. At the same time, interaction of Gp120 with TLR9 was observed in pDC [208]. Another way that HIV can evade IFN therapy is by significantly reducing the level of transcription of many antiviral ISGs, including, i.a., XAF1, OAS1, and AXL, thereby bypassing IFN treatment [209,210]. According to some studies, prolonged exposure to type I IFNs in HIV-infected patients in the chronic phase of infection has been associated with deleterious hyperimmune induction contributing to disease progression [211,212]. Although IFN therapy has been recognized as a promising approach to the treatment of HIV, the role played by type I IFN in the course and control of HIV infection is still unknown. Many questions remain unanswered despite intensive research in this field [196].

#### 2.3.6. Epstein–Barr Virus (EBV)

The Epstein–Barr virus (EBV) is a DNA-type virus belonging to the herpesvirus family that causes a persistent infection that can last a lifetime in a latent state in infected body cells in most of the world’s population. EBV infection activates most cell signaling pathways, leading to the production of type I IFN, which inhibits EBV infection and virus-induced transformation of B cells [213,214,215]. The EBV virus is the first virus with oncogenic potential discovered and its main target cells are B lymphocytes. The EBV virus infects, in particular, epithelial cells of the pharynx and oral cavity through infection with saliva, where EBV implication occurs, which then leads to uncontrolled proliferation and killing of B lymphocytes [216] and natural killer cells, [217,218]. In most cases, EBV infection is asymptomatic, which can cause infectious mononucleosis and lead to serious diseases and carcinogenesis of many malignant tumors. It is associated with cancers of epithelial origins, such as nasopharyngeal cancer, and cancers originating from cells of the hematopoietic and lymphatic systems, such as Burkitt and Hodgkin lymphoma [219,220,221,222]. During the lytic replication and latency phase of the EBV virus, it triggers several viral gene expression models, thanks to which the virus bypasses the reaction of the human immune system. In addition, the virus triggers the induction of many cellular signaling pathways, including TLR pathways [223,224], regulatory factor IRF3 and IRF7, and cellular factor NF-κβ [225], resulting in the formation of type I IFN [224]. However, EBV lytic replication and EBV-depleted B cells show increased resistance to the antiproliferative effects of type I IFNs. This is due to the replication of latency proteins such as EBV-2 nuclear antigen (EBNA-2) and EBV membrane proteins (LMP1), which are considered to be oncoproteins [223,224,225].

Production of type I IFN by EBNA-2 in lymphoblastoid cells (LCL) results in priority expression of type I IFN and induction of STAT1. The LMP1 protein, probably through IRF7 and NF-κβ, activates the secretion of type I IFN, which activates the phosphorylation of STAT1 and IRF7, thanks to which it is possible to determine the course of EBV latency [226,227,228]. IFN-α secretion in EBV infection inhibits EBV early antigen activation when EBV replication is activated by superinfection, but does not inhibit antigen activation in cells undergoing lytic replication [217,226,227].

Data from the literature indicate that IFN-β, unlike IFN-α, does not inhibit EBV early antigen activation in cells unless replication is induced by butyrate [228,229,230], but inhibits early EBV excretion from the throat and mouth in immunocompromised individuals [231,232,233].

Other data indicate that IFN-β also inhibits EBV transformation [234,235]. The EBV envelope protein BGLF2 binds to the tyrosine kinase Tyk2, inhibiting the phosphorylation of STAT1 and STAT3 proteins necessary for immune control of EBV and for maintaining EBV latency [236]. It also blocks IFN signaling type I by counteracting the propensity of IFN-α and IFN-β to inhibit EBV reactivation.

IFN-α is released by infected cells that are triggered by viral DNA and are dependent on endosomal TLR9 signaling. pDCs express TLR7 and TLR9 and function as the primary sources of IFN-α release immediately after EBV infection [237,238,239]. 

#### 2.3.7. Severe Acute Respiratory Syndrome Coronavirus 2 (SARS-CoV-2)

The human severe acute respiratory syndrome coronavirus 2 (SARS-CoV-2) first appeared in December 2019 and has caused many cases of infection and death among the entire world population. The newly discovered SARS-CoV-2 shares about 80% of the nucleotide sequence of its predecessor, the severe respiratory syndrome coronavirus (SARS-CoV) from two decades ago. Interestingly, over 30% of SARS-CoV virus proteins have an inhibitory effect on the immune response mediated by type I IFN [240,241,242,243,244,245]. Studies by Lei et al. indicate that the SARS-CoV-2 virus induces the type I INF response and that therapies with the participation of IFN-β effectively inhibit the replication of the SARS-CoV-2 virus [246]. As with other viruses, the first line of defense against the SARS-CoV-2 virus is host innate immunity, of which the type I IFN system is a part and which is closely related to PAMPs via the PRR [247,248]. However, the exact signaling pathway resulting in the inhibition of IFN production is not yet fully understood. Previous studies indicate that SARS-CoV-2 infection stimulates delayed production of IFN, which suggests that it weakens the immune response as a result of the development of many strategies to antagonize the antiviral response of the human body [249,250,251].

The research team of Kumari et al. [252] showed that the SARS-CoV-2 virus is much more sensitive to type I IFN pretreatment than the SARS-CoV virus. Their study used isolated cell lines (Vero E6) to produce type I IFN that have been successfully used in exogenous therapy [252]. Moreover, infection of respiratory epithelial cells (Calu-3) against type I IFN resulted in reduced SARS-CoV-2 replication compared with SARS-CoV [253]. Studies have shown that there are significant genetic differences in the structure of both epidemic virus strains, which may affect their ability to modulate type I IFN responses. The final study showed that the results in Calu-3 cells are consistent with those of Vero E6 cells and indicate the sensitivity of the SARS-CoV-2 virus to the initial treatment with type I IFN. In addition, research on the SARS-CoV-2 virus shows that type I IFN therapy is promising; however, our knowledge about the novel SARS-CoV-2 coronavirus needs to be verified in terms of operation, infection, and disease caused by the virus [254,255,256,257,258]. Studies conducted by two independent teams [259,260] indicate that the SARS-CoV virus proteins, i.e., ORF3b, Nsp1, and ORF6, not only inhibit the expression of IFN-β in infected cells but additionally prevent ISRE activation and ISG expression in the presence of exogenous recombinant IFNs, indicating that these factors may block the IFNAR signaling pathway. Type III IFNs have been identified as potentially significant in the context of SARS-CoV-2 infection [261]. Clinical trials on IFN-λ for the treatment of patients with COVID-19 are being conducted with the aim of exerting antiviral effects without inflammatory effects. However, given the still relatively low level of knowledge in this area, IFN treatment should be carefully evaluated. Understanding the exact role of type I and III IFNs in SARS-CoV-2 infection should be explored to facilitate future treatment of COVID-19 patients.

### 2.4. The Role of Interferons in the Course of Bacterial Infections

**Figure 3:**
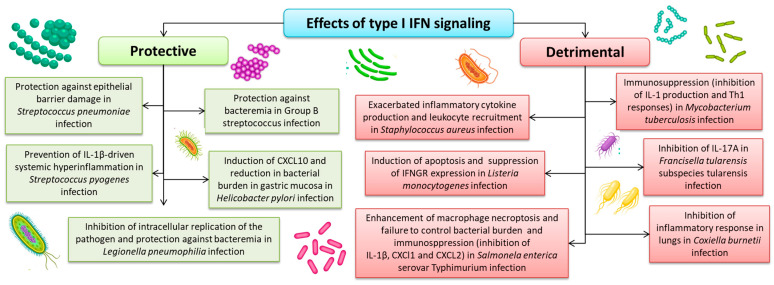
Effects of type I IFN signaling in bacterial infection (based on [18,262,263,264]).

Type I IFN plays an important role in the course of bacterial infections. Despite this, its role in the course of bacterial infections is not entirely clear and requires extensive research [18]. The signaling of type I IFNs to bacterial host infections is quite diverse, which may be influenced by many factors, e.g., site, route of infection, level of bacterial replication and expression, as well as virulence factors or immune response avoidance strategies. A differentiated effect depending on the route of infection has also been observed in many disease processes [262]. Recent studies indicate that type I IFNs may act as critical immune-promoting cytokines during infection with several species of bacteria. Moreover, the mechanism of action of type I IFNs during bacterial infections may have not only protective functions but also be detrimental to the body’s immune mechanisms (Figure 3). The reasons for such diverse roles of type I IFN in bacterial infections are still unclear and require many interdisciplinary studies.

Based on research, scientists have shown that bacteria trigger the production of type I IFN mainly by recognizing their nucleic acids or lipopolysaccharide (LPS), which is one of the components of the cell wall [263,264,265]. As a result of the recognition of these PAMPs, innate immune response mechanisms are activated. The activation pathways of IFN induction mechanisms have been best described for IFN-β, which, according to studies, is among the first to be produced during infection, along with IFN-α4, and is also the driving force of other type I IFN genes [18,266,267]. Researchers suggest that the induction of IFN-β by bacterial DNA is an extremely complex process and may involve many different pathways. One of the most common methods of inducing IFN-β is the cyclic GMP-AMP synthase with a cytosolic DNA sensor [268,269,270,271]. This mechanism has been described for bacterial infections caused by such pathogens such as *Francisella novicida*, group B streptococcus (GBS) (*Streptococcus agalactiae*), *Legionella pneumophila*, *Listeria monocytogenes*, *Mycobacterium tuberculosis* or *Neisseria gonorrhoeae* [272,273,274,275,276,277,278]. Moreover, the recognition of bacterial DNA (through unmethylated DNA containing the CpG motif) may also occur through the endosomal receptor TLR9, which as a consequence of activation of signal transduction pathways, contributes to the production of IFN-β [279,280,281]. Research in recent years has shown that bacterial RNA may also be a key inducer of IFN-β. This is due to a short and conserved sequence found in bacterial 23S rRNA that is recognized by mouse TLR13. Activation of this receptor contributes to the induction of the Myd88- and IRF5-dependent pathways, which consequently influence the production of IFN-β [282,283,284,285]. In the case of the human body, TLR8 is used as a bacterial RNA sensor, but the exact mechanism of its activation leading to the induction of IFN-β is still not fully understood [286,287,288].

The mechanism of IFN-β induction by LPS occurs after its binding to TLR4 and internalization into the endosome [289,290]. Subsequently, TRIF-dependent activation of TBK1 kinase causes phosphorylation of the transcription factor IRF3 to stimulate transcription of the IFN-β gene. Endosomal signaling is also involved in the induction of IFN-β by TLR2 in response to Gram-positive bacteria, although this mechanism appears to be limited to specific immune cells and/or pathogens [18]. Although the pathways that cause IFN-β induction by bacteria are relatively well understood, further research is needed to assess the importance of individual pathways for overall IFN-β production throughout the body rather than within cells.

## 3. Materials and Methods

### 3.1. Search Strategy, Study Selection, and Data Extraction

**Figure 4:**
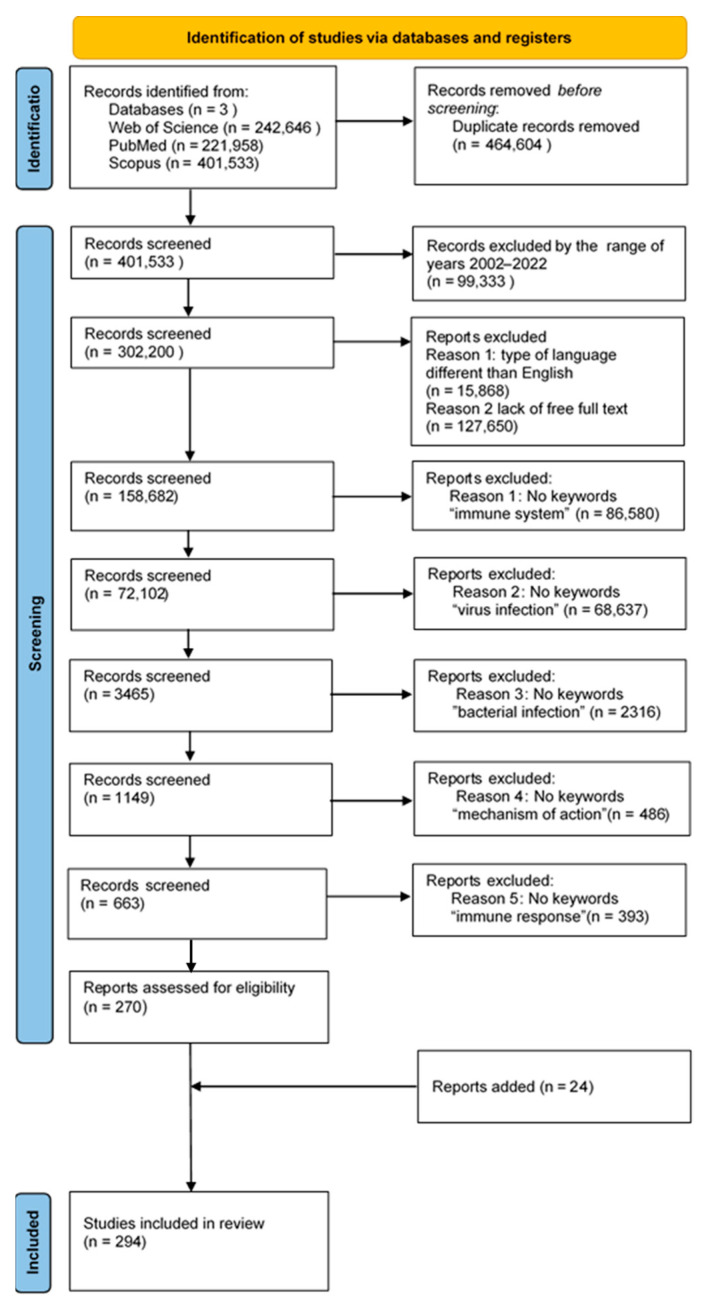
Search strategy, study selection, and data extraction according to the PRISMA statement.

The analysis of the literature was carried out in the Web of Science, PubMed and Scopus databases, where the search for available articles was made based on the following keywords: “interferon”, “immune system”, “virus infection”, “bacterial infection”, “mechanism of action”, “immune response”. The time range was set to 2002–2022, using filters related to the type of articles (review, systematic review, clinical trials, meta-analysis). The articles produced by this search were assessed for access to the full version. Then, the selected articles were assessed for inclusion in the publication. Duplicates were rejected at each stage of the analysis among the found articles. In the end, 276 articles meeting the criteria were included. The procedure is presented below (Figure 4).

### 3.2. Bioinformatics Analyses of the Amino Acid Sequences of IFNs

For bioinformatics analyses, the amino acid sequences deposited in the UniProt database [291] were used. The IFNs identification numbers and their amino acid sequences are provided in Supplementary Materials Table S1. These sequences were used to carry out further bioinformatics analyses. The sequence length and molecular weight of individual interleukins were taken from the UniProt database. The determination of the isoelectric point of the tested interleukins and their amino acid compositions was carried out using the IPC isoelectric point calculator software available online [292]. The analysis of the second-order structure of the IFNs was carried out using the NetSurfP-3.0 program available online [293].

### 3.3. Analysis of the Amino Acid Sequence Identity of IFNs

The amino acid sequences of the selected interleukins from the UniProt databases (Supplementary Materials Table S1) were used for the analyses. The amino acid sequences of individual IFNs were compared with each other using the Clustal Omega program available in Reference [294]. The results of the analyses are presented as the percentage of identical amino acids in the analyzed amino acid sequences and are collected in Supplementary Materials Table S2.

## 4. Conclusions

The human body’s immune system can generate interferons, which are substances that aid in stimulating and regulating the immune response to viral infections and specific cancers. Interferons have been subject to extensive study and have been employed in medical treatments, including antiviral therapies, cancer treatment, and immunomodulation. Despite their benefits, interferon-based therapies can produce significant side effects, such as flu-like symptoms, fatigue, depression, and hematologic abnormalities, and may exhibit varying effectiveness based on the disease type, patient, and disease stage. Interferons function by activating genes that participate in signaling pathways to assist the immune system in fighting infections, such as by activating immune cells and regulating antigen presentation to T cells. Despite numerous studies, there is still much to learn about interferons, and researchers continue to make progress in understanding their mechanisms of action. Interdisciplinary studies are crucial in optimizing the use of interferons in therapeutic therapies, particularly in the context of combining them with other drugs to improve therapeutic outcomes and patient survival. This review is intended to inspire scientists to explore new research directions and to uncover unknown interactions between interferons and pathogens.

## Figures and Tables

**Table 2:** Characteristics of the occurrence of interferon isoforms.

Name	Isoform	Protein ID	Characteristic	Reference
IFN-α6	Alpha-6X1A	P23229-2	259-297: Missing 1084-1130:RYDDSVPRYHAVRIRKEEREIKDEKYIDNLEKKQWITKWNENESYS → NKKDHYDATYHKAEIHAQPSDKERLTSDA	[33]
	Alpha-6X1B	P23229-3	259-297: Missing	
	Alpha-6X2A	P23229-4	215-258: Missing 1084-1130:SRYDDSVPRYHAVRIRKEEREIKDEKYIDNLEKKQWITKWNENESYS → NKKDHYDATYHKAEIHAQPSDKERLTSDA	
	Alpha-6X2B	P23229-5	215-258: Missing	
	Alpha-6X1X2A	P23229-6	1084-1130:SRYDDSVPRYHAVRIRKEEREIKDEKYIDNLEKKQWITKWNENESYS → NKKDHYDATYHKAEIHAQPSDKERLTSDA	
	7	P23229-7	1-114: Missing 215-258: Missing 1084-1130:SRYDDSVPRYHAVRIRKEEREIKDEKYIDNLEKKQWITKWNENESYS → NKKDHYDATYHKAEIHAQPSDKERLTSDA	
	9	P23229-9	918-932: Missing 1084-1130:SRYDDSVPRYHAVRIRKEEREIKDEKYIDNLEKKQWITKWNENESYS → NKKDHYDATYHKAEIHAQPSDKERLTSDA	
IFN-α7	2	P36544-2	18-18: H → HGKATASPPSTPPWDPGHIPGASVRPAPGP	[33]
	3	P36544-3	81-102: SWTDHYLQWNVSEYPGVKTVRF → AYSRVPATSMYAGFPLMCSTAN 103-502: Missing	
IFN-α16	IFI 16B	Q16666-2	444-499: Missing	[38]
	IFI 16C	Q16666-3	444-555: Missing	
	4	Q16666-6	128-183: Missing	
IFN-λ4	p170	K9M1U5-2	123-179:LELARPGSSRKVPGAQKRRHKPRRADSPRCRKASVVFNLLRLLTWELRLAAHSGPCL → VSDGRAPPPLSPASFSASSGPRRAPALCQSVLLSGKTHPDRSRVLWVS	[50]
	p131	K9M1U5-3	75-122: Missing	
	p107	K9M1U5-4	51-122: Missing	

**Table 3:** Effector pathways of the antiviral response in which interferons are involved.

Name	Characteristics	Reference
Mx antiviral protein pathway	Mx proteins are evolutionarily conserved large dynamin-like GTPases, and GTPase activity is required for their antiviral activity; They are key mediators of innate antiviral resistance induced in cells by type I (α/β) and type III (λ) interferons (IFNs); Inhibits a wide range of viruses by blocking the early stage of the replication cycle.	[84,85,86,87]
Ribonuclease L pathway targeting 2’,5’-oligoadenylate synthetase	The 2′,5′-oligoadenylate synthetase (OAS)/RNase L pathway is a component of innate immunity that responds to a pathogen-associated molecular pattern (dsRNA) to degrade viral and cellular RNAs, thereby blocking the infection; The 2-5A molecule is a unique ligand for RNase L and is believed to be a trigger for the signaling of innate antiviral immunity mechanisms by activating RNase L.	[88,89]
Protein kinase R pathway	Protein Kinase R (PKR) is one of the best-characterized IFN effector molecules; Mediates responses by phosphorylation of protein substrates and promotes signal transduction pathways to maintain homeostasis; Involved in activating immune mechanisms and promoting apoptosis in cells.	[90,91,92]
Pathway similar to ISG15	ISG15 is a member of the ubiquitin family, which includes ubiquitin and ubiquitin-like modifiers; This protein is strongly induced by type I interferons; ISG15 may compete with ubiquitin for ubiquitin-binding sites on the protein, thereby indirectly regulating protein degradation; This protein acts as a cytokine and can also interact with various intracellular protein partners.	[93,94,95]

## Data Availability

Materials necessary for preparing this publication are included as Supplementary Materials. More information can be obtained from the corresponding author of this publication.

## Supplementary Materials

The following supporting information can be downloaded at: https://www.mdpi.com/article/10.3390/ijms241210115/s1.

## Abbreviations

CMV	cytomegalovirus
DCs	dendritic cells
dsRNA	double-stranded RNA
EBV	Epstein–Barr virus
GAF	gamma-interferon activation factor
GAS	gamma ray sequences
GBP	guanylate binding proteins
HBLV	Human B-lymphotropic virus
HCV	Hepatitis C Virus
HHV-6	human herpesvirus type 6
HHV-7	human herpesvirus type 7
HIV	human immunodeficiency virus
HSV	herpes simplex virus
IFITM	interferon-induced transmembrane proteins
IFN-ɛ	interferon epsilon
IFNs	interferons
IFN-α	interferon alpha
IFN-β	interferon beta
IFN-γ	interferon gamma
IFN-δ	interferon delta
IFN-ζ	interferon zeta
IFN-κ	interferon kappa
IFN-ν	interferon nu
IFN-τ	interferon tau
IFN-χ	interferon chi
IFN-ω	interferon omega
ISGs	interferon-stimulated genes
ISRE	interferon regulatory factor
JAK2	Janus kinase 2
LCMV	lymphocytic choriomeningitis virus
LCMV	Lymphocytic Choriomeningitis Virus
MDA5	melanoma-differentiation-associated gene 5
MHC	Major histocompatibility complex
NOS2	nitric oxide synthase 2
OAS	oligoadenylate synthases
pDCs	plasmacytoid dendritic cells
RIG-I	retinoic-acid-inducible protein I
SARS-CoV	coronavirus
STAT	Signal transducer and activator of transcription
TRIM	triremembered motif proteins
TYK2	tyrosine kinase 2
VZV	herpes zoster
