# Supplementary material for: Immunomodulatory Role of Interferons in Viral and Bacterial Infections
Source: Int J Mol Sci. 2023 Jun 14;24(12):10115. doi: 10.3390/ijms241210115 (PMC10298684; doi:10.3390/ijms241210115)
Supplement: Supplementary file 1 [file ijms-24-10115-s001.zip › ijms-2417656-supplementary.pdf]

Supplementary Materials—Table S1: Interferon Sequences

**Protein ID**	**Sequence**
P01562	MASPFALLMVLVVLSCKSSCSLGCDLPETHSLDNRRTLMLLAQMSRISPSSCLMDRHDFGFPQ<br>EEFDGNQFQKAPAISVLHELIQQIFNLFTTKDSSAAWDEDLLDKFCTELYQQLNDLEACVMQEE<br>RVGETPLMNADSILAVKKYFRRITLYLTEKKYSPCAWEVVRAEIMRSLSLSTNLQERLRRKE
P01563	MALTFALLVALLVLSCKSSCSVGCDLPQTHSLGSRRTLMLLAQMRRISLFSCLKDRHDFGFPQEE<br>FGNQFQKAETIPVLHEMIQQIFNLFSTKDSSAAWDETLLDKFYTELYQQLNDLEACVIQGVGVT<br>ETPLMKEDSILAVRKYFQRITLYLKEKKYSPCAWEVVRAEIMRSFSLSTNLQESLRSKE
P05014	MALSFSLLMAVLVLSYKSICSLGCDLPQTHSLGNRRALILLAQMGRISHFSCLKDRHDFGFPEEE<br>FDGHQFQKAQAISVLHEMIQQTFNLFSTEDSSAAWEQSLLEKFSTELYQQLNDLEACVIQEVGV<br>EETPLMNEDSILAVRKYFQRITLYLTEKKYSPCAWEVVRAEIMRSLSFSTNLQKRLRRKD
P01569	MALPFVLLMALVVLNCKSICSLGCDLPQTHSLSNRRTLMIMAQMGRISPFSCLKDRHDFGFPQ<br>EEFDGNQFQKAQAISVLHEMIQQTFNLFSTKDSSATWDETLLDKFYTELYQQLNDLEACMMQ<br>EVGVEDTPLMNVDSILTVRKYFQRITLYLTEKKYSPCAWEVVRAEIMRSFSLSANLQERLRRKE
P23229	MAAAGQLCLLYLSAGLLSRLGAAFNLDTREDNVIRKYGDPGSLFGFSLAMHWQLQPEDKRLLL<br>VGAPRAEALPLQRANRTGGLYSCDITARGPCTRIEFDNDADPTSESKEDQWMGVTVQSQGPG<br>GKVVTCAHRYEKRQHVNTKQESRDIFGRCYVLSQNLRIEDDMDGGDWSFCDGRLRGHEKFGS<br>CQQGVAATFTKDFHYIVFGAPGTYNWKGIVRVEQKNNTFFDMNIFEDGPYEVGGETEHDESL<br>VPVPANSYLGLLFLTSVSYTDPDQFVYKTRPPREQPDTFPDVMMNSYLGFSLDSGKGIVSKDEIT<br>FVSGAPRANHSGAVVLLKRDMKSAHLLPEHIFDGEGLASSFGYDVAVVDLNKDGWQDIVIGA<br>PQYFDRDGEVGGAVYVYMNQQGRWNNVKPIRLNGTKDSMFGIAVKNIGDINQDGYPDIAV<br>GAPYDDLGKVFIYHGSANGINTKPTQVLKGISPYFGYSIAGNMDLDRNSYPDVAVGSLSDSVTIF<br>RSRPVINIQKTITVTPNRIDLRQKTACGAPSGICLQVKSCFEYTANPAGYNPSISIVGTLEAEKERR<br>KSGLSSRVQFRNQGSEPKYTQELTLKRQKQKVCMEETLWLQDNIRDKLRPIPITASVEIQEPSSR<br>RRVNSLPEVLPILNSDEPKTAHIDVHFLKEGCGDDNVCNSNLKLEYKFCTREGNQDKFSYLPIQK<br>GVPELVLKDQKDIALEITVTNSPSNPRNPTKDGDDAHEAKLIATFPDTLTYSAYRELRAFPEKQLS<br>CVANQNGSQADCELGNPFKRNSNVTFYLVLSTTEVTFDTPDLDINLKLETTSNQDNLAPITAKA<br>KVVIELLLSVSGVAKPSQVYFGGTVVGEQAMKSEDEVGSLIEYEFRVINLGKPLTNLGTATLNIQ<br>WPKEISNGKWLLYLVKVESKGLEKVTCEPQKEINSLNLTESHNSRKKREITEKQIDDNRKFSLFAE<br>RKYQTLNCSVNVNCVNIRCPLRGLDSKASLILRSRLWNSTFLEEYSKLNYLDILMRAFIDVTAAAE<br>NIRLPNAGTQVRVTVFPSKTVAQYSGVPWWIILVAILAGILMLALLVFILWKCGFFKRSRYDDSV<br>PRYHAVRIRKEEREIKDEKYIDNLEKKQWITKWNENESYS
P36544	MRCSPGGVWLALAASLLHVSLQGEFQRKLYKELVKNYNPLERPVANDSQPLTVYFSLSLLQIMD<br>VDEKNQVLTTNIWLQMSWTDHYLQWNVSEYPGVKTVRFPDGQIWKPDILLYNSADERFDAT<br>FHTNVLVNSSGHCQYLPPGIFKSSCYIDVRWFPFDVQHCKLKFGSWSYGGWSLDLQMQEADI<br>SGYIPNGEWDLVGIPGKRSERFYECCKEPYPDVTFTVTMRRRTLYYGLNLLIPCVLISALALLVFLL<br>PADSGEKISLGITVLLSLTVFMLLVAEIMPATSDSVPLIAQYFASTMIIVGLSVVVTVIVLQYHHHD<br>PDGGKMPKWTRVILLNWCAWFLRMKRPGEDKVRPACQHKQRRCSLASVEMSAVAPPPASN<br>GNLLYIGFRGLDGVHCVPTPDSGVVCGRMACSPTHDEHLLHGGQPPEGDPDLAKILEEVRYIA<br>NRFRCQDESEAVCSEWKFAACVVDRLCLMAFSVFTIICTIGILMSAPNFVEAVSKDFA
P32881	MALTFYLLVALVVLSYKSFSSLGCDLPQTHSLGNRRALILLAQMRRISPFSCLKDRHDFEFPQEEF<br>DDKQFQKAQAISVLHEMIQQTFNLFSTKDSSAALDETLLDEFYIELDQQLNDLESCVMQEVGVI<br>ESPLMYEDSILAVRKYFQRITLYLTEKKYSSCAWEVVRAEIMRSFSLSINLQKRLKSKE
P01566	MALSFSLLMAVLVLSYKSICSLGCDLPQTHSLGNRRALILLGQMGRISPFSCLKDRHDFRIPQEEF<br>DGNQFQKAQAISVLHEMIQQTFNLFSTEDSSAAWEQSLLEKFSTELYQQLNDLEACVIQEVGV<br>EETPLMNEDSILAVRKYFQRITLYLIERKYSPCAWEVVRAEIMRSLSFSTNLQKRLRRKD
P01570	MALPFALMMALVVLSCKSSCSLGCNLSQTHSLNNRRTLMLMAQMRRISPFSCLKDRHDFEFP<br>QEEFDGNQFQKAQAISVLHEMMQQTFNLFSTKNSSAAWDETLLEKFYIELFQQMNDLEACVI<br>QEVGVEETPLMNEDSILAVKKYFQRITLYLMEKKYSPCAWEVVRAEIMRSLSFSTNLQKRLRRK

	D
Q16666	MGKKYKNIVLLKGLEVINDYHFRMVKSLLSNDLKLNLKMREEYDKIQIADLMEEKFRGDAGLGKLIKIFEDIPTLEDLAETLKKEKLKVKGPALSRKRKKEVDATSPAPSTSSTVKTEGAEATPGAQKRKKSTKEKAGPKGSKVSEEQTQPPSPAGAGMSTAMGRSPSPKTSLSAPPNSSSTENPKTVAKCQVTPRRNVLQKRPVIVKVLSTTKPFEYETPEMEKKIMFHATVATQTQFFHVKVLNTSLKEKFNGKKIIIISDYLEYDSLLEVNEESTVSEAGPNQTFEVPNKIINRAKETLKIDILHKQASGNIVYGVFMLHKKTVNQKTTIYEIQDDRGKMDVVGTGQCHNIPCEEGDKLQLFCFRLRKKNQMSKLISEMHSFIQIKKKTNPRNNDPKSMKLPQEQRQLPYPSEASTTFPESHLRTPQMPPTTPSSSFFTKKSEDTISKMNDFMRMQILKEGSHFPGPFMTSIGPAESHPHTPQMPPSTPSSSFLTTKSEDTISKMNDFMRMQILKEGSHFPGPFMTSIGPAESHPHTPQMPPSTPSSSFLTTLKPRLKTEPEEVSIEDSAQSDLKEVMVLNATESFVYEPKEQKKMFHATVATENEVFRVKVFNIDLKEKFTPKKIIAIANYVCRNGFLEVYPFTLVADVNADRNMEIPKGLIRSASVTPKINQLCSQTKGSFVNGVFEVHKKNVRGEFTYYEIQDNTGKMEVVVHGRLTTINCEEGDKLKLTCFELAPKSGNTGELRSVIHSHIKVIKTRKNKKDILNPDSSMETSPDFFF
P01571	MALSFSLLMAVLVLSYKSICSLGCDLPQTHSLGNRRALILLAQMGRISPFSCLKDRHDFGLPQEEFDGNQFQKTQAISVLHEMIQQTFNLFSTEDSSAAWEQSLLEKFSTELYQQLNNLEACVIQEVGMEETPLMNEDSILAVRKYFQRITLYLTEKKYSPCAWEVVRAEIMRSLSFSTNLQKILRRKD
P01568	MALSFSLLMAVLVLSYKSICSLGCDLPQTHSLGNRRALILLAQMGRISPFSCLKDRHDFGFPQEEFDGNQFQKAQAISVLHEMIQQTFNLFSTKDSSATWEQSLLEKFSTELNQQLNDLEACVIQEVGVEETPLMNVDSILAVKKYFQRITLYLTEKKYSPCAWEVVRAEIMRSFSLSKIFQERLRRKE
P01574	MTNKCLLQIALLLCFSTTALSMSYNLLGFLQRSSNFQCQKLLWQLNGRLEYCLKDRMNFDIPEEIKQLQQFQKEDAALTIYEMLQNIFAIFRQDSSSTGWNETIVENLLANVYHQINHLKTVLEEKLEKEDFTRGKLMSSLHLKRYYGRILHYLKAKEYSHCAWTIVRVEILRNFYFINRLTGYLRN
P05000	MALLFPLLAALVMTSYSPVGSLGCDLPQNHGLLSRNTLVLLHQMRRISPFLCLKDRRDFRFPQEMVKGSQLQKAHVMSVLHEMLQQIFSLFHTERSSAAWNMTLLDQLHTGLHQQLQHLETCLLQVVGEGESAGAISSPALTLRRYFQGIRVYLKEKKYSDCAWEVVRMEIMKSLFLSTNMQERLRSKDRDLGSS
Q86WN2	MIIKHFFGTVLVLLASTTIFSLDLKLIIFQQRQVNQESLKLLNKLQTLSIQQCLPHRKNFLLPQKSLSPQQYQKGHTLAILHEMLQQIFSLFRANISLDGWEENHTEKFLIQLHQQLEYLEALMGLEAEKLSGTLGSDNLRLQVKMYFRRIHDYLENQDYSTCAWAIVQVEISRCLFFVFSLTEKLSKQGRPLNDMKQELTTEFRSPR
Q9P0W0	MSTKPDMIQKCLWLEILMGIFIAGTLSLDCNLLNVHLRRVTWQNLRHLSSMSNSFPVECLRENIAFELPQEFLQYTQPMKRDIKKAFYEMSLQAFNIFSQHTFKYWKERHLKQIQIGLDQQAEYLNQCLEEDKNENEDMKEMKENEMKPSEARVPQLSSLELRRYFHRIDNFLKEKKYSDCAWEIVRVEIRRCLYYFYKFTALFRRK
P01579	MKYTSYILAFQLCIVLGSLGCYCQDPYVKEAENLKKYFNAGHSDVADNGTLFLGILKNWKEESDRKIMQSQIVSFYFKLFKNFKDDQSIQKSVETIKEDMNVKFFNSNKKKRDDFEKLTNYSVTDLNVQRKAIHELIQVMAELSPAAKTGKRKRSQMLFRGRRASQ
Q8IU54	MAAAWTVVLVTLVLGLAVAGPVPTSKPTTTGKGCHIGRFKSLSPQELASFKKARDALEESLKLKNWSCSSPVFPGNWDLRLLQVRERPVALEAELALTLKVLEAAAGPALEDVLDQPLHTLHHILSQLQACIQPQPTAGPRPRGRLHHWLHRLQEAPKKESAGCLEASVTFNLFRLLTRDLKYVADGNLCLRTSTHPEST
Q8IZJ0	MKLDMTGDCTPVLVLMAAVLTVTGAVPVARLHGALPDARGCHIAQFKSLSPQELQAFKRAKDALEESLLLKDCRCHSRLFPRTWDLRQLQVRERPMALEAELALTLKVLEATADTDPALVDVLDQPLHTLHHILSQFRACIQPQPTAGPRTRGRLHHWLYRLQEAPKKESPGCLEASVTFNLFRLLTRDLNCVASGDLCV
Q8IZI9	MTGDCMPVLVLMAAVLTVTGAVPVARLRGALPDARGCHIAQFKSLSPQELQAFKRAKDALEESLLLKDCKCRSRLFPRTWDLRQLQVRERPVALEAELALTLKVLEATADTDPALGDVLDQPLHTLHHILSQLRACIQPQPTAGPRTRGRLHHWLHRLQEAPKKESPGCLEASVTFNLFRLLTRDLNCVASGDLCV
K9M1U5	MRPSVWAAVAAGLWVLCTVIAAAPRRCLLSHYRSLEPRTLAAAKALRDRYEEEALSWGQRNC

	SFRPRRDPPRPSSCARLRHVARGIADAQAVLSGLHRSELLPGAGPILELLAAAGRDVAACLELAR PGSSRKVPGAQKRRHKPRRADSPRCRKASVVFNLLRLLTWELRLAAHSGPCL

P01562

Your protein (peptide) has 189 amino acids.

Ala 12	Phe 9	Val 9	Cys 7	Ser 18	Asp 11	Lys 9
Met 8	Gly 4	Trp 2	Asn 6	Thr 9	Glu 15	Arg 12
Pro 7	Ile 7	Leu 27	Gln 10	Tyr 4	Sec 0	His 3

Protein mass: 21725.06114 Da

P01563

Your protein (peptide) has 189 amino acids.

Ala 10	Phe 11	Val 10	Cys 6	Ser 15	Asp 10	Lys 9
Met 10	Gly 5	Trp 2	Asn 7	Thr 11	Glu 13	Arg 11
Pro 6	Ile 8	Leu 24	Gln 13	Tyr 5	Sec 0	His 3

Protein mass: 21942.39254 Da

P05014

Your protein (peptide) has 189 amino acids.

Ala 12	Phe 11	Val 9	Cys 5	Ser 19	Asp 8	Lys 9
Met 6	Gly 6	Trp 2	Asn 5	Thr 8	Glu 16	Arg 11
Pro 4	Ile 9	Leu 26	Gln 13	Tyr 5	Sec 0	His 5

Protein mass: 21808.04644 Da

P01569

Your protein (peptide) has 189 amino acids.

Ala 10	Phe 11	Val 10	Cys 6	Ser 15	Asp 10	Lys 9
Met 10	Gly 5	Trp 2	Asn 7	Thr 11	Glu 13	Arg 11
Pro 6	Ile 8	Leu 24	Gln 13	Tyr 5	Sec 0	His 3

Protein mass: 21942.39254 Da

P23229

Your protein (peptide) has 1130 amino acids.

Ala 65	Phe 45	Val 80	Cys 21	Ser 80	Asp 72	Lys 72
Met 15	Gly 81	Trp 15	Asn 66	Thr 62	Glu 69	Arg 62
Pro 56	Ile 64	Leu 102	Gln 47	Tyr 41	Sec 0	His 15

Protein mass: 126605.50084 Da

P36544

Your protein (peptide) has 502 amino acids.

Ala 30	Phe 22	Val 44	Cys 18	Ser 37	Asp 27	Lys 20
Met 14	Gly 31	Trp 13	Asn 16	Thr 21	Glu 23	Arg 23
Pro 32	Ile 26	Leu 56	Gln 18	Tyr 18	Sec 0	His 13

Protein mass: 56449.43874 Da

P32881

Your protein (peptide) has 189 amino acids.

Ala 11	Phe 12	Val 10	Cys 4	Ser 20	Asp 11	Lys 11
Met 6	Gly 3	Trp 1	Asn 4	Thr 7	Glu 15	Arg 10
Pro 4	Ile 10	Leu 27	Gln 13	Tyr 7	Sec 0	His 3

Protein mass: 21989.33804 Da

P01566

Your protein (peptide) has 189 amino acids.

Ala 11	Phe 10	Val 9	Cys 5	Ser 19	Asp 8	Lys 8
Met 6	Gly 6	Trp 2	Asn 6	Thr 7	Glu 15	Arg 13
Pro 5	Ile 11	Leu 26	Gln 14	Tyr 5	Sec 0	His 3

Protein mass: 21835.15914 Da

P01570

Your protein (peptide) has 189 amino acids.

Ala 12	Phe 12	Val 9	Cys 6	Ser 17	Asp 7	Lys 11
Met 12	Gly 3	Trp 2	Asn 9	Thr 8	Glu 15	Arg 11
Pro 5	Ile 7	Leu 23	Gln 13	Tyr 4	Sec 0	His 3

Protein mass: 22062.62614 Da

Q16666

Your protein (peptide) has 785 amino acids.

Ala 33	Phe 38	Val 46	Cys 8	Ser 68	Asp 30	Lys 89
Met 29	Gly 37	Trp 0	Asn 37	Thr 63	Glu 63	Arg 28
Pro 57	Ile 44	Leu 53	Gln 29	Tyr 14	Sec 0	His 19

Protein mass: 88255.54994 Da

P01571

Your protein (peptide) has 189 amino acids.

Ala 11	Phe 10	Val 8	Cys 5	Ser 19	Asp 7	Lys 9
Met 7	Gly 6	Trp 2	Asn 7	Thr 9	Glu 15	Arg 10
Pro 5	Ile 10	Leu 27	Gln 14	Tyr 5	Sec 0	His 3

Protein mass: 21728.05614 Da

P01568

Your protein (peptide) has 189 amino acids.

Ala 11	Phe 12	Val 10	Cys 5	Ser 19	Asp 7	Lys 11
Met 6	Gly 6	Trp 2	Asn 6	Thr 8	Glu 15	Arg 10
Pro 5	Ile 10	Leu 25	Gln 14	Tyr 4	Sec 0	His 3

Protein mass: 21741.12644 Da

P01574

Your protein (peptide) has 187 amino acids.

Ala<br>8	Phe<br>10	Val<br>5	Cys<br>5	Ser<br>11	Asp<br>5	Lys<br>12
Met<br>5	Gly<br>6	Trp<br>3	Asn<br>13	Thr<br>10	Glu<br>13	Arg<br>11
Pro<br>1	Ile<br>12	Leu<br>30	Gln<br>12	Tyr<br>10	Sec<br>0	His<br>5

Protein mass: 22293.88484 Da

P05000

Your protein (peptide) has 195 amino acids.

Ala<br>10	Phe<br>8	Val<br>11	Cys<br>4	Ser<br>19	Asp<br>7	Lys<br>8
Met<br>10	Gly<br>10	Trp<br>2	Asn<br>4	Thr<br>8	Glu<br>10	Arg<br>14
Pro<br>6	Ile<br>5	Leu<br>33	Gln<br>14	Tyr<br>4	Sec<br>0	His<br>8

Protein mass: 22319.05604 Da

Q86WN2

Your protein (peptide) has 208 amino acids.

Ala<br>7	Phe<br>13	Val<br>7	Cys<br>3	Ser<br>15	Asp<br>6	Lys<br>13
Met<br>5	Gly<br>7	Trp<br>2	Asn<br>8	Thr<br>11	Glu<br>14	Arg<br>10
Pro<br>5	Ile<br>13	Leu<br>36	Gln<br>21	Tyr<br>5	Sec<br>0	His<br>7

Protein mass: 24414.41284 Da

Q9P0W0

Your protein (peptide) has 207 amino acids.

Ala 8	Phe 13	Val 6	Cys 6	Ser 12	Asp 8	Lys 17
Met 9	Gly 3	Trp 4	Asn 11	Thr 6	Glu 20	Arg 15
Pro 6	Ile 12	Leu 24	Gln 13	Tyr 9	Sec 0	His 5

Protein mass: 25218.24074 Da

P01579

Your protein (peptide) has 166 amino acids.

Ala 9	Phe 11	Val 9	Cys 3	Ser 13	Asp 10	Lys 21
Met 5	Gly 7	Trp 1	Asn 10	Thr 6	Glu 9	Arg 8
Pro 2	Ile 9	Leu 14	Gln 10	Tyr 7	Sec 0	His 2

Protein mass: 19348.30394 Da

Q8IU54

Your protein (peptide) has 200 amino acids.

Ala 21	Phe 5	Val 13	Cys 5	Ser 13	Asp 6	Lys 11
Met 1	Gly 11	Trp 4	Asn 4	Thr 14	Glu 12	Arg 12
Pro 15	Ile 3	Leu 33	Gln 8	Tyr 1	Sec 0	His 8

Protein mass: 21898.41234 Da

Q8IZJ0

Your protein (peptide) has 200 amino acids.

Ala<br>21	Phe<br>6	Val<br>13	Cys<br>8	Ser<br>8	Asp<br>12	Lys<br>8
Met<br>4	Gly<br>8	Trp<br>2	Asn<br>2	Thr<br>13	Glu<br>10	Arg<br>16
Pro<br>13	Ile<br>3	Leu<br>34	Gln<br>10	Tyr<br>1	Sec<br>0	His<br>8

Protein mass: 22288.01304 Da

Q8IZI9

Your protein (peptide) has 196 amino acids.

Ala<br>21	Phe<br>5	Val<br>13	Cys<br>8	Ser<br>8	Asp<br>11	Lys<br>8
Met<br>3	Gly<br>9	Trp<br>2	Asn<br>2	Thr<br>12	Glu<br>10	Arg<br>17
Pro<br>13	Ile<br>3	Leu<br>34	Gln<br>10	Tyr<br>0	Sec<br>0	His<br>7

Protein mass: 21706.37244 Da

K9M1U5

Your protein (peptide) has 179 amino acids.

Ala<br>29	Phe<br>2	Val<br>10	Cys<br>7	Ser<br>14	Asp<br>5	Lys<br>5
Met<br>1	Gly<br>10	Trp<br>4	Asn<br>2	Thr<br>3	Glu<br>8	Arg<br>27
Pro<br>14	Ile<br>3	Leu<br>25	Gln<br>3	Tyr<br>2	Sec<br>0	His<br>5

Protein mass: 19674.97994 Da
